# Medical Defence

**Published:** 1914-07-18

**Authors:** 


### THE PROFESSION OF MEDICINE.

### Medical Defence.

The annual reports of the London and Counties Medical Protection Society and of the Medical Defence Union for the past year are, as usual, records of steady progress and increasing usefulness. As in previous years, the details of their work in protecting harassed practitioners against blackmail and a hundred other forms of annoyance are very similar in the two cases. Suffice it that even a cursory glance through the list of cases successfully defended—and defeat is almost unknown to both of these societies—should convince every medical man in England that it is his duty to himself and his neighbour to join one or other of them the moment he qualifies, and to pay his subscription regularly throughout his life. Union is indeed strength in the practice of medicine, and those who know by experience the pitfalls and enemies lying in wait for unwary practitioners are most emphatic about the value for money obtained for a subscription to these societies. The young practitioner will find it better worth while to take this for granted and to join at once than to wait for some bitter experience to teach him the lesson; added to which he gets the extra advantage of being able to sleep comfortably in his bed at night, secure against at least one of the calamities that before now have wrecked a promising medical career.

Some two or three years ago, as is well known, the two societies diverged somewhat in respect of the lines of their financial arrangements. This fact inevitably makes for comparisons between the systems on which they respectively work and the results from a business point of view. Formerly both societies, for an annual premium of ten shillings and an entrance fee of the same amount, insured their members against the whole cost of their medical defence. (We are purposely leaving this last term vague, as in both cases there were qualifying clauses of much the same effect). They did not undertake to indemnify their members for damages found against them or for the costs of a successful opponent. To supply insurance against these possibilities the two societies adopted different schemes. The Defence Union arranged with the Yorkshire Insurance Company to insure any members who might desire it against this extra risk up to £2,000 for a premium of seven shillings and sixpence, and up to £2,500 for a premium of nine shillings. The Medical Protection Society, on the other hand, increased the annual subscription from ten shillings to a pound, and in return assured members against adverse verdicts up to £2,000 in any one case. It will be appreciated that the advantages of the Defence Union plan are the optional character of the extra insurance, the freedom from risk to the accumulated resources, and the cheaper rate at which it is provided; while the merits of the Protection Society's way are that such profits as may be derived from the insurance are retained for the future benefit of members and go to strengthen the society's position; and that a larger measure of protection is afforded.

The time is now approaching when some estimate will be possible of the relative popularity and the relative success of these two different systems. Let us take first the bolder policy of the Protection Society, and examine its working. Out of every subscription of twenty shillings, eleven shillings is allotted to a general account, against which is charged the whole of the ordinary expenditure of the society. The income of this fund is therefore one shilling per member per annum in excess of that of the ordinary income of the Defence Union. The account shows an annual surplus which has varied in the last three years from £230 to £810. Such surpluses, and also all the entrance fees, are invested to the credit of a reserve fund account. This fund and the balance in hand of the general account amount to £6,123, and represent the savings on the ordinary work of the Society during the twenty-two years of its existence. The membership of the Society is about 5,000, so that this reserve may be roughly placed at £1 4s. 6d. per head.

Then there is a separate fund called the insurance account; this is credited with the remaining nine shillings of each subscription, and with dividends on investments of this fund, and debited with the expenditure incurred by adverse verdicts. This risk, in excess of £2,000 and up to £22,000, is reinsured with an outside company at an annual cost of about eighteenpence per member. The fund shows a very large annual surplus, and since it was established three years ago has grown to £5,543. The result, therefore, of the revised scale of premium and benefits adopted three years ago has been very greatly to increase the profits and the accumulated resources of the Society. Nor has it, apparently, had very much effect upon the popularity of the Society. It is true that the new membership, which reached a maximum of 439 in 1907, was only 270 in 1909, 332 in 1910, and 302 in 1911; but it rose to 350 in 1912 and to 391 in 1913. Unquestionably the Council of the Society have been justified in their policy, which created some misgiving when it was inaugurated.

The Medical Defence Union has, as we have noted, stuck to the old ten shillings subscription and not undertaken any responsibility for the consequences of adverse verdicts against its members. Like the junior union, its income and expenditure accounts always show a balance on the right side; but it is to be noted that entrance fees and dividends on investments are treated as ordinary income. Deducting these items there would have been a loss in 1913. The accumulated investments of the Union, together with its cash balances, come to a total of about £5,090. The membership is 8,660, so that the reserves at the end of 1913 are equal to just under twelve shillings per member, or less than half the proportion in the London and Counties Society. (This represents part only of the potential resources of the Union, since there is also a guarantee fund standing at £11,539.) It would not be fair to draw or to suggest the inference that the Defence Union is in an inferior position of financial stability to that of the Protection Society, or that it offers less attraction to new entrants. There are many other points in the management which would require to be taken into consideration before any such discrimination would be justified. To mention one only, it is probable that the prestige of the Defence Union is greater than that of the junior and smaller organisation; and prestige counts for a great deal in medical defence. Indeed, the protection gained by the mere announcement that a defence society is behind any particular practitioner is perhaps the most valuable single item of the benefits which membership confers. The Defence Union, too, spends a good deal of money on persecuting impostors and on other work which benefits the whole profession impartially: this, in itself, is a strong claim on the gratitude and support of practitioners.

However, it is apparent that the policy of the London and Counties Society during the last three years has "paid"; and there are indications to be found in the report of the Defence Union that this fact has not been lost upon its Council. A significant passage in the report of the Finance Committee states an opinion that the time may soon arrive to increase the subscription to one guinea. Since the Union pays its way very comfortably on a subscription of ten shillings, it is obvious that this sentence must mean the assumption of extra liabilities beyond those now incurred—in other words, of some approach to the system of the Protection Society. The Union is so well served by its secretary that it may seem ungracious to criticise the rapid rise in his remuneration; nevertheless, it seems to have risen about 40 or 50 per cent. in the last five years or so, and to be on a scale which by comparison with that of the Protection Society seems slightly extravagant.

